# Inhibitory Role of Growth Hormone in the Induction and Progression Phases of Collagen-Induced Arthritis

**DOI:** 10.3389/fimmu.2018.01165

**Published:** 2018-05-25

**Authors:** Ricardo Villares, Gabriel Criado, Yasmina Juarranz, Mercedes Lopez-Santalla, Eva M. García-Cuesta, José M. Rodríguez-Frade, Javier Leceta, Pilar Lucas, José Luis Pablos, Carlos Martínez-A, Marina I. Garin, Rosa P. Gomariz, Mario Mellado

**Affiliations:** ^1^Department of Immunology and Oncology, Centro Nacional de Biotecnología/CSIC, Madrid, Spain; ^2^Inflammatory and Autoimmune Diseases Group, Instituto de Investigación Hospital 12 de Octubre (i+12), Madrid, Spain; ^3^Departamento de Biología Celular, Facultad de Biología, Universidad Complutense de Madrid, Madrid, Spain; ^4^Division of Hematopoietic Innovative Therapies, Centro de Investigaciones Energéticas Medioambientales y Tecnológicas (CIEMAT) and Centro de Investigación Biomédica en Red de Enfermedades Raras (CIBERER-ISCIII), Madrid, Spain; ^5^Advanced Therapy Unit, Instituto de Investigación Sanitaria Fundación Jiménez Díaz (IIS-FJD/UAM), Madrid, Spain

**Keywords:** collagen-induced arthritis, growth hormone, Th17/Th1 cells, cytokines, immune regulation

## Abstract

Evidence indicates an intimate connection between the neuroendocrine and the immune systems. A number of *in vitro* and *in vivo* studies have demonstrated growth hormone (GH) involvement in immune regulation. The GH receptor is expressed by several leukocyte subpopulations, and GH modulates immune cell proliferation and activity. Here, we found that sustained GH expression protected against collagen-induced arthritis (CIA); in GH-transgenic C57BL/6 (GHTg) mice, disease onset was delayed, and its overall severity was decreased. The anti-collagen response was impaired in these mice, as were inflammatory cytokine levels. Compared to control arthritic littermates, immunized GHTg mice showed significantly lower RORγt (retinoic acid receptor-related orphan receptor gamma 2), IL-17, GM-CSF, IL-22, and IFNγ mRNA expression in draining lymph nodes, whereas there were no differences in IL-21, IL-6, or IL-2 mRNA levels. Data thus suggest that Th17/Th1 cell plasticity toward a pathological phenotype is reduced in these mice. Exogenous GH administration in arthritic DBA/1J mice reduced the severity of established CIA as well as the inflammatory environment, which also shows a GH effect on arthritis progression. These results indicate that GH prevents inflammatory joint destruction in CIA. Our findings demonstrate a modulatory GH role in immune system function that contributes to alleviating CIA symptoms and underlines the importance of endocrine regulation of the immune response.

## Introduction

Growth hormone (GH) is secreted by the anterior pituitary in vertebrates. Although initially defined as a major stimulant in somatic growth control, it is in fact a pleiotropic hormone that affects many physiological functions. GH regulates bone and muscle mass (1, 2), carbohydrate, fat, and protein metabolism (3), sexual maturation (4), and insulin resistance (5). Several *in vitro* and *in vivo* studies also demonstrate GH involvement in immune regulation, and the GH receptor is expressed by several leukocyte subpopulations (6). GH mediates thymic development (7), promotes T cell engraftment in severe combined immunodeficiency mice (8), improves B cell responses and antibody production (9, 10), and modulates NK cell (11) and macrophage activity (12) as well as *in vivo* Th1/Th2 and humoral immune responses (13). Some reports describe beneficial effects of GH administration in autoimmunity. GH administration and neutralization of TNFα reduce mucosal inflammation in experimental colitis (14); by altering tolerization mechanisms such as the cytokine environment, macrophage polarization, activation of the suppressor T cell population, and Th17 cell plasticity, GH also reduces type I diabetes development (15).

Rheumatoid arthritis (RA) is the most prevalent inflammatory autoimmune disease worldwide. Its main clinical feature is chronic inflammation in joints, associated with bone and cartilage destruction (16). The RA spectrum and disease progression are governed by immune, genetic, and environmental factors (17). Its origin nonetheless lies in an inappropriate inflammatory reaction derived from deregulation of the adaptive and/or innate branches of the immune response. During RA development, there is active proliferation of endothelial cells and synovial fibroblasts; the synovium displays features of chronic inflammation, including massive leukocyte infiltration of innate (macrophages, NK, and dendritic cells; DC) and adaptive (CD4^+^ T and B cells) immune response cells (16).

Using collagen-induced arthritis (CIA) as a model of RA, we observed that GH transgenic (GHTg) mice were protected against disease development, whose onset was delayed and severity reduced. Our data demonstrated an inhibitory role of GH in the induction phase of the disease. The anti-collagen response was severely impeded in GHTg mice, as was the synthesis of inflammatory cytokines, suggesting impairment of Th17/Th1 cell plasticity toward a pathological phenotype. GH also modulated the CIA progression phase, shown by reduced severity of established disease in collagen-immunized DBA/1J mice following exogenous GH administration. Our data demonstrate that GH administration ameliorates CIA symptoms pointing out an important role of this hormone tuning the immune response. Altogether, our results underline the interrelationship between the endocrine and the immune systems that regulate the immune response and support a potential use of endogenous endocrine mediators for the treatment of inflammatory and autoimmune diseases.

## Materials and Methods

### Mice

Mice transgenic for bovine GH (bGH) under the control of the phosphoenolpyruvate carboxykinase promoter on a C57BL/6J background (18) were maintained by continuous backcrosses on C57BL/6J females. 35 transgenic mice (GHTg) and 33 control littermates (10–14 weeks old) were used, with matched sex ratios in each experiment. DBA/1J mice (50 males) were obtained from Charles River Laboratories International. Three OVA-specific TCR-transgenic mice (OT-II) were donated by Dr. C. Ardavín (Centro Nacional de Biotecnología, Madrid, Spain). Mice were handled according to national and European Union guidelines, and experiments were approved by the Comité Ético de Experimentación Animal, Centro Nacional de Biotecnología/CSIC and the Regional Government (PROEX 250-16).

### CIA Induction and Treatment

Two-month-old GHTg mice, control littermates, or DBA/1J mice were immunized intradermally (i.d.) at the tail base with an emulsion of chicken type II collagen (CII) in citrate buffer and Freund’s complete adjuvant (19). Arthritis was assessed daily by scoring each limb on a 0–4 scale, where 0 = normal, 1 = erythema and mild swelling confined to the tarsals or ankle joint, 2 = erythema and mild swelling extending from the ankle to the tarsals, 3 = erythema and moderate swelling extending from the ankle to metatarsal joints, and 4 = erythema and severe swelling encompassing the ankle, foot, and digits, or ankylosis of the limb, yielding a maximum score of 16 per mouse. In some cases, on appearance of the first signs of CIA (score ~2 = day 0), affected DBA/1J mice were separated into two groups; one group received a daily subcutaneous (s.c.) rhGH injection (2 µg/ml, 200 µl, Genotonorm, Pfizer) until day 9 and the other received only PBS as control. Clinical scores (Cs_t_) were re-evaluated daily.

### Histochemistry

At the end of the experiments, the score was monitored and paws removed, fixed in 4% formalin, decalcified with 500 mM EDTA (Sigma) at 4°C, and paraffin embedded. Sections (7-µm thick) were hematoxylin/eosin-stained. When needed, safranin O/light green and fast green or TRAP staining were carried out using a leukocyte acid phosphatase staining kit (Sigma-Aldrich). Sections were analyzed in an Olympus BX51 microscope equipped with a digital camera (Olympus DP70).

### Cell Purification and Flow Cytometry

To prepare single-cell suspensions, spleens and lymph nodes (LNs) were harvested and minced on a 40-µm nylon mesh in RPMI 1640 medium (Lonza) supplemented with 10% fetal bovine serum (FBS), 2 mM l-glutamine, and 50 µg/ml penicillin/streptomycin. Erythrocytes were lysed with VersaLyse (Beckman Coulter). Single-cell suspensions of lymphoid organs or blood leukocytes were prepared and blocked with anti-CD16/32 (BD Pharmingen) to impede nonspecific Fc-mediated antibody binding. Samples were stained with antibody conjugates by a standard procedure, using FITC anti-CD25, FITC anti-CD11b, SPRD (Spectral Red) anti-Gr1 (Pharmingen), SPRD anti-CD4 and PE-Cyan5 anti-F4/80 (BM8) (eBiosciences), FITC anti-CD8, -CD69, -CD45, APC (allophycocyanin) anti-B220 (Beckman Coulter), and PE (phycoerythrin) anti-CD86 (BioLegend). FoxP3, IL-10, IL-17, GM-CSF, IFNγ, and TNFα expression was determined after permeabilization and intracellular staining with a PE-labeled antibody (FoxP3 staining set; eBiosciences), FITC-labeled antibody to IL-10 (JES516E3, Pharmingen) and to GM-CSF (MP1-22E9, eBiosciences), and PE-labeled antibody to IL17 (TC11-18H10, Pharmingen), PE-Cyan7-labeled antibody to TNFα (MP6-xt3, eBiosciences), and APC-labeled antibody to IFNγ (XMG1.2, R&D Systems).

For *in vitro* characterization of CD4^+^ and CD8^+^ T cell subpopulations in the LNs of GHTg and control mice, 1 × 10^6^ cells/ml were cultured (4 h, 37°C, 5% CO_2_) in RPMI supplemented with 10% FBS, 50 ng/ml PMA (Sigma), and 500 ng/ml ionomycin (Sigma). For intracellular staining, 2.5 µM monensin (Sigma) was added after 60 min of culture. Cells were then fixed with 2% paraformaldehyde and permeabilized with 0.2% Saponin (Sigma) prior to be stained with the appropriated antibodies.

For the analysis of LNs in GH-treated arthritic DBA/1J mice and controls, 5 × 10^6^ cells/ml were cultured (5 h, 37°C, 5% CO_2_) in RPMI supplemented with 10% FBS, 5 ng/ml PMA (Sigma), 500 ng/ml ionomycin (Sigma), 25 µM TAPI-1 (Enzo), GolgiStop and GolgiPlug (BD Pharmingen). Cells were fixed and stained according to the manufacturer’s instructions. For intracellular staining of FoxP3, we employed the FoxP3/transcription factor staining buffer set (eBiosciences) and we used the Cytokine BD kit BD Pharmingen for the rest of cytokines evaluated.

When required, cells isolated from LNs were cultured (48 h, 37°C, 5% CO_2_) in plates coated with anti-CD3 (100 ng/ml, 4 h, 37°C; clone 145-2C11, BioLegend). IFNγ and IL-7 levels were determined in culture supernatants using the ELISA MAX sets (BioLegend).

When necessary, murine naïve B cells were purified from spleen using mouse pan-B Dynabeads (Invitrogen). Batches with >95% purity were activated with 5 µg/ml goat anti-mouse IgM Ab (Jackson ImmunoResearch; 6 h, 37°C) or 5 µg/ml LPS (lipopolysaccharide from *Escherichia coli* serotype 055:B5; Sigma; 6 h, 37°C), alone or with exogenous recombinant human GH (rhGH, 5 µg/ml, Genotonorm; Pfizer). Stained samples as above were analyzed on a flow cytometer (Cytomics FC 500; Beckman Coulter). FACS data were analyzed using the FlowJo (FlowJo LLC) and CytoSpec (Germany) software.

### Bone Marrow Dendritic Cell (BM-DC) Differentiation

Bone marrow dendritic cell from GHTg or control littermates were obtained from bone marrow cell suspensions treated with erythrocyte lysis buffer and cultured in 150-mm Petri dishes in complete RPMI 1640 medium supplemented with 10% FCS and 20 ng/ml recombinant granulocyte–macrophage colony-stimulating factor (PeproTech) (20). Immature BM-DC were collected on day 8 and purity evaluated by flow cytometry using anti-CD11c-FITC and -MHCII-PE Ab (BD Pharmingen). Batches of >95% purity were used for maturation with LPS (1 µg/ml, 12 h, 37°C).

### *In Vitro* Induction of Immune Synapse (IS) Formation Between DC and CD4 T Cells

Immune synapse formation between mouse BM-DC and OT-II CD4^+^ T cells was induced as reported (21, 22). Briefly, mature BM-DC (1 µg/ml LPS, 12 h, 37°C) were loaded with OVA_323–339_-peptide (5 µM, 30 min, 37°C; GenScript) and mixed in complete medium (RPMI 1640, 10% FCS) with CD4^+^ T cells (1:5 DC:CD4^+^ T cells) from OT-II mouse spleen and LNs, purified with a mouse T cell negative isolation kit (MACS; Miltenyi Biotec; T cell purity was routinely >97%). Cells were centrifuged (120 × g, 2 min) in a conical tube and incubated in complete medium (15–30 min, 37°C) to foster IS formation.

### T Cell Proliferation

OT-II CD4^+^ T cells were labeled with CellTrace Violet (0.5 µM, 30 min, 37°C; Molecular Probes) and added to plates containing OVA peptide-loaded BM-DC (3 × 10^4^ DC/6 × 10^4^ T cells). Cells were co-cultured in RPMI 1640 with 10% FCS (24, 48, or 72 h), and proliferation determined by flow cytometry using dye dilution evaluation in a Gallios flow cytometer (Beckman Coulter). The percentage of dividing cells was calculated using the FlowJo. When needed, 5 µg/ml rhGH was added.

### Enzyme-Linked Immunoassay

Microtiter plates were coated with chicken type II collagen in PBS (5 µg/ml; 90 min, 37°C). After blockade of free protein-binding sites with 0.5% BSA/PBS, plates were incubated with serial dilutions of serum samples from immunized mice, followed by peroxidase-labeled subclass-specific rabbit anti-mouse antisera (Dako).

### Semiquantitative Real-Time PCR

cDNA sequences were obtained from the GenBank database. PCR primers were designed from the cDNA sequences using Primer-BLAST software (Table 1). RNA (5 µg) was used for reverse transcription. cDNA was obtained by SuperScript II reverse transcriptase (Invitrogen). cDNA was then amplified by PCR using Power SYBR Green PCR Master Mix (Applied Biosystems) and 0.3 µM of primers. Triplicate samples were quantified using the ABI Prism HT7900 sequence detection system (Applied Biosystems). For relative quantification, we used the equation 2^−ΔCt^. Each sample was normalized with β-actin (∆Ct) and the respective basal levels in the spleen of wild-type (wt) mice (ΔΔCt).

**Table 1:** PCR primer sequences.

Gene name	Genebank accession number	Sequence position	Primers	Sequence
β-Actin	NM007393	694–831	for rev	5′-AGAGGGAAATCGTGCGTGAC-3′ 5′-CAATAGTGATGACCTGGCCGT-3′
Tbx21	NM009322	474–574	for rev	5′-CACTAAGCAAGGACGGCGAA-3′ 5′-CCACCAAGACCACATCCACA-3′
RORγt	NM011281	808–908	for rev	5′-GAAGCTGGGAGCTATGCAGG-3′ 5′-TGGCTACGATGCAGCAAGGAG-3′
Foxp3	AY357713	929–1029	for rev	5′-TGCTGATGGGAGGAGATGTCT-3′ 5′-TTTCTTTCAGGGACAGCCTGTT-3′
IFNγ	K00083	929–1029	for rev	5′-TGCTGATGGGAGGAGATGTCT-3′ 5’-TTTCTTTCAGGGACAGCCTGTT-3′
IL-2	NM008366	252–352	for rev	5′-GAAACTCCCCAGGATGCTC A-3′ 5′-GCCGCAGAGGTCCAAGTTC-3′
IL-10	NM010548	113–218	for rev	5′-TGACTGGCATGAGGATCAGC-3′ 5′-AGTCCGCAGCTCTAGGAGCA-3′
IL-17A	NM010552	338–398	for rev	5′-GAAGCTCAGTGCCGCCA-3′ 5′TTCATGTGGTGGTCCAGCTTT-3′
IL-21	NM021782	510–581	for rev	5′-AAGATTCCTGAGGATCCGAGAA-3′ 5′-GCATTCGTGAGCGTCTATAGTGTC-3′
IL-22	NM016971	204–271	for rev	5′-TACATCGTCAACGCACCTTT-3′ 5′-CGGACGTCTGTGTTGTTAT-3′
IL-6	NM031168	187–327	for rev	5′-GAGGATACCACTCCCAACAGACC-3′ 5′-AAGTGCATCATCGTTGTTCATACA-3′
TGFβ1	NM011577	1697–1790	for rev	5′-GGAGAGCCCTGGATACCAAC-3′ 5′-CAACCCAGGTCCTTCCTAAA-3′
CCL21a	NM001193667	250–378	for rev	5′-ATCCCGGCAATCCTGTTCTC-3′ 5′-GGGGCTTTGTTTCCCTGGG-3′
CCL20	NM001159738	107–224	for rev	5′-CTGCTGGCTCACCTCTGCA-3′ 5′-CATCGGCCATCTGTCTTGTG-3′
CXCL12	NM021704	244–344	for rev	5′-AGCCAACGTCAAGCATCTGA-3′ 5′-TCGGGTCAATGCACACTTGT-3′

*Primer sequences employed for PCR detection of several factors involved in the differentiation and function of Th17, Th1, or Treg subsets, as well as for β-actin (housekeeping gene)*.

### Statistical Analysis

Statistical analyses were performed using GraphPad Prism 5.0 software (GraphPad). Unpaired Student’s *t*-test, Mann–Whitney *U*-test, and two-way ANOVA were used as indicated (in all cases, *p ≤ 0.05, **p ≤ 0.01, ***p ≤ 0.001).

## Results

### GHTg Mice Are Protected Against CIA

**Figure 1:**
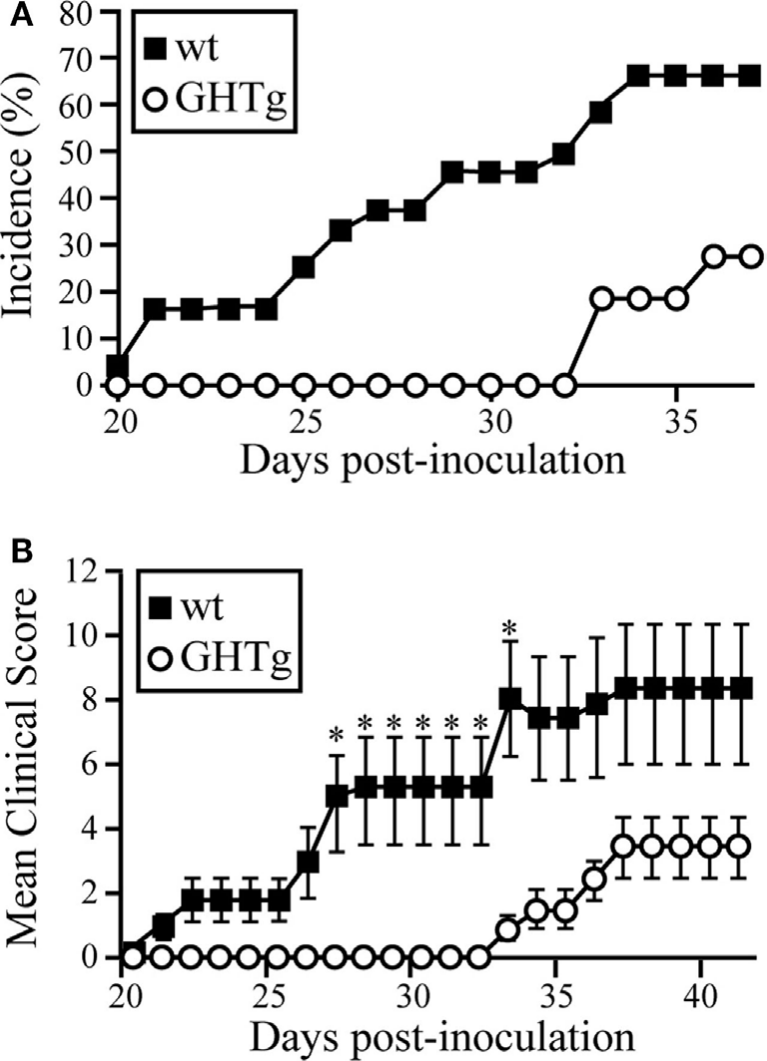
GHTg mice are resistant to collagen-induced arthritis. One representative experiment is shown of three performed (*n* = 11 mice/group). **(A)** Cumulative incidence of arthritic symptoms in wild-type (wt) and GHTg mice after immunization with chicken type II collagen. **(B)** Clinical scores in affected wt and GHTg mice. Data shown as mean ± SEM (Student’s *t*-test, *p ≤ 0.05). Two-way ANOVA shows significant differences for time (*p* = 0.0217) and genotype (*p* < 0.0001) factors.

**Figure 2:**
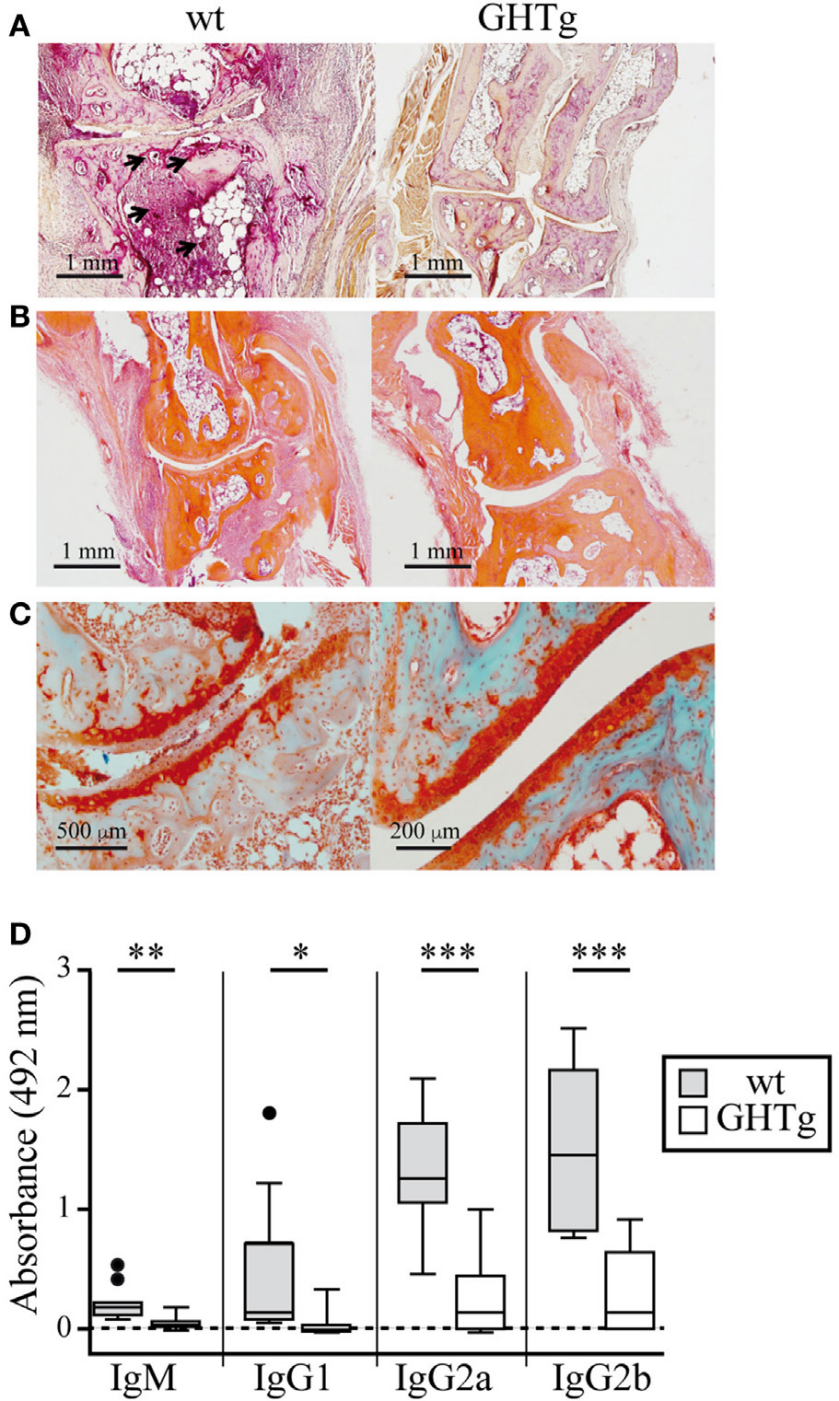
Histological comparison in cartilage and bone of collagen-immunized wild-type (wt) and GHTg mice. Representative images at day 30 post-inoculation. **(A)** Acid phosphatase reaction in formaldehyde-fixed paraffin embedded joint sections showing osteoclasts (arrows). **(B)** Hematoxylin/eosin staining shows pannus only in the wt sample. **(C)** Safranin-light green staining shows no evidence of cartilage degradation in the GHTg mouse sample. **(A,B)** Original magnification, 2.5× (bar, 1 mm); **(C)** 5× (left; bar, 500 µm), 10× (right; bar, 200 µm). **(D)** Isotype-specific ELISA to detect anti-chicken collagen II antibodies in sera from the immunized mice. Individual values are shown for sera diluted 1/200, except for IgG2b (1/1,000). Tukey boxplot showing median and 1.5 interquartile range. Student’s *t*-test (*p ≤ 0.05, **p ≤ 0.01, ***p ≤ 0.001).

To analyze the effect of GH in immune response regulation, we tested its effect in a murine model of CIA on a mouse strain transgenic for bGH under the control of the rat phosphoenolpyruvate carboxykinase promoter; these mice showed constant circulating GH (~5 μg/ml) (18). The CIA model allows the study of the priming phase of the disease, which consists of activation of the collagen type II-specific immune response as well as the inflammatory effector phase, characterized by local inflammation, and cartilage and joint destruction (23). GHTg mice were partially protected, and developed arthritis at a lower incidence compared to wt arthritic control littermates (GHTg vs. wt, 0 vs. 45% at day 30, 20 vs. 70% at day 35). The GHTg mice also showed delayed disease onset (GHTg, day 33 vs. wt, day 21) (Figure 1A) concomitant with a marked decrease in CIA severity (Figure 1B), as shown by evaluation of the incidence and clinical score. Consistent with these results, histopathological examination at day 30 highlighted a marked reduction in synovial inflammation, pannus formation, erosion of articular cartilage in GHTg vs. control arthritic mice, and presence of osteoclasts in the joints of wt mice (Figures 2A–C).

In accordance with a previous report (19), we detected low IgG1 levels in wt arthritic mice, while IgG2 was the predominant isotype in late disease phases (day 25). IgG1 and IgG2 responses were nonetheless equally reduced in GHTg mice (Figure 2D). The lesser severity of CIA symptoms in GHTg mice was associated with changes in anti-collagen type II antibody levels. Anti-collagen IgM and IgG antibody levels were significantly lower in GHTg mice relative to wt arthritic mice, which suggests that GH triggered protection during the induction phase of the disease (Figure 2D).

**Figure 3:**
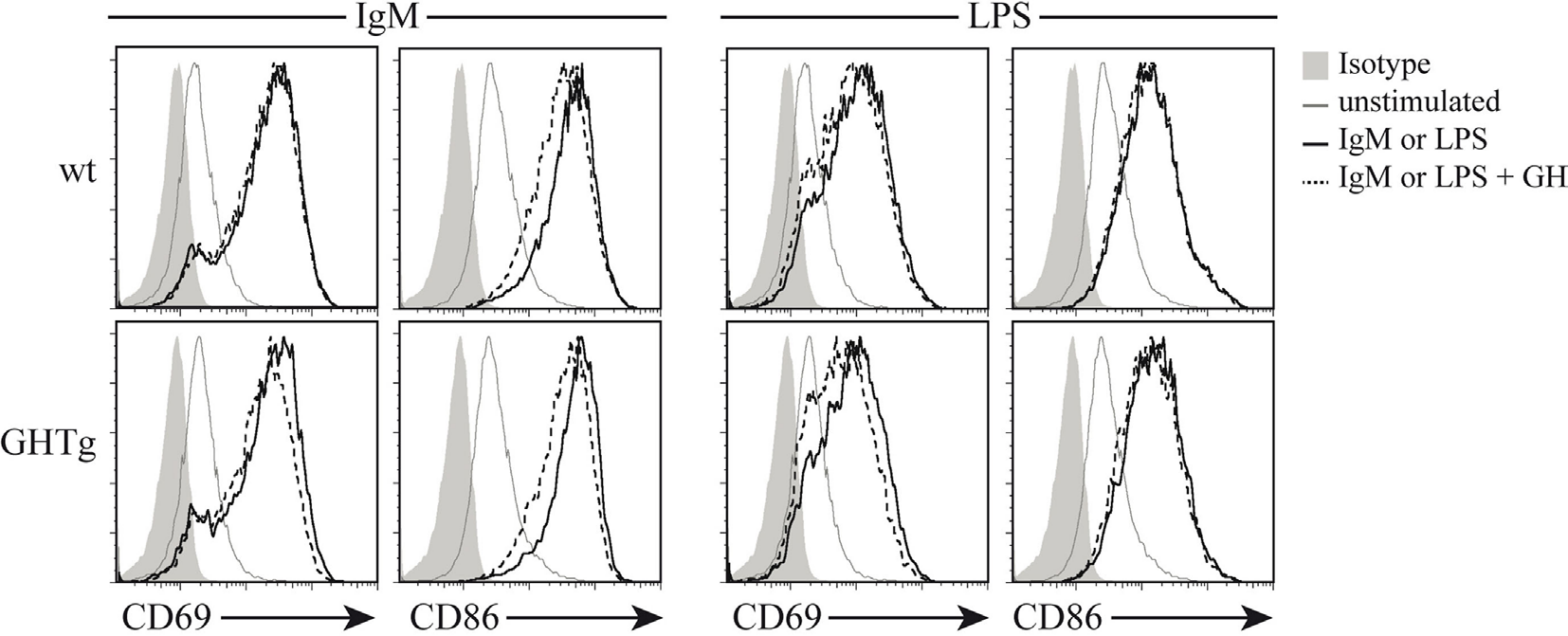
Growth hormone (GH) does not alter B-cell activation *in vitro*. Splenic B cells from wild-type (wt) and GHTg mice were unstimulated or incubated (6 h, 37°C) with anti-IgM antibody or LPS alone or with exogenous rhGH (5 µg/ml). The activation level was determined using anti-CD69 and anti-CD86 mAb in flow cytometry. One representative experiment of three is shown.

As reported (13), flow cytometry analysis showed that in basal conditions compared to controls, GHTg mice had a significantly higher proportion of B220^+^ cells in LNs and reduced percentage of CD4^+^ and CD8^+^ T cells, whereas no variations were found in the percentage of immune cell populations either in blood or in spleen (Table 2). When evaluated different subpopulations of T cells we did not detect differences (Table 3). Previous reports suggest a role for GH in modulating the immune response (10, 12, 13, 15); we thus characterized immune cell subsets in blood and peripheral LNs from collagen-immunized GHTg mice and wt littermates. Flow cytometry analysis showed that, compared to controls, immunized GHTg mice had a significantly larger number of B220^+^ cells in peripheral LNs (Table 4). In agreement with a previous report (15), GH did not affect *in vitro* B cell activation by either anti-mouse IgM antibodies or LPS (Figure 3), which rules out intrinsic *in vitro* activation defects of GHTg mouse B cells. The immunized GHTg mice also had significant lower percentage of CD4^+^IFNγ^+^ and of CD8^+^IFNγ^+^ cells and a marked tendency to higher proportion of CD4^+^FoxP3^+^ cells compared to control littermates (Table 5). These data suggest differences in T cell polarization between both groups of mice.

**Table 2:** Distribution of cell populations in wild-type (wt) and GHTg mice.

	Spleen		Peripheral lymph nodes (LNs)		Blood	
	wt	GHTg	wt	GHTg	wt	GHTg
CD8^+^	20.4 ± 5.8	17.6 ± 4.5	**32.6 ± 0.9**	21.5 ± 1.7**	15.7 ± 0.5	11.3 ± 0.4
CD4^+^	17.4 ± 2.1	12.8 ± 2.1	**32.3 ± 1.0**	25.1 ± 1.5*	16.3 ± 1.9	13.6 ± 2.0
B220^+^	68.6 ± 2.2	66.1 ± 2.1	**28.6 ± 2.2**	48.8 ± 0.6***	46.7 ± 1.8	53.8 ± 2.4
CD11b^+^	14.9 ± 1.0	16.0 ± 0.2	10.7 ± 2.8	13.2 ± 3.2	29.8 ± 6.4	20.7 ± 2.2
GR1^+^	1.3 ± 0.3	2.6 ± 0.5	0.3 ± 0.1	0.6 ± 0.3	8.4 ± 0.8	11.4 ± 1.7

*Distribution of cell populations in blood, spleen, and peripheral LNs of 12-week-old wt and GHTg mice. Numbers correspond to the percentage of positive cells ± SD indicated (*n* = 3). Bold numbers highlight significant differences between GHTg and control mice, as indicated*.

Student’s t-test, *p < 0.05, **p < 0.01, ***p < 0.001.

**Table 3:** Distribution of CD3^+^/CD4^+^ and CD3^+^/CD8^+^ cell populations in non-immunized wild-type (wt) and GHTg mice.

		Peripheral lymph nodes (LNs)			
		wt	GHTg
CD4^+^	Foxp3^+^	10.75 ± 1.48	16.83 ± 2.17
	IFNγ^+^	21.10 ± 6.93	21.40 ± 1.85
	IL-17^+^	0.335 ± 0.205	1.113 ± 0.340
CD8^+^	IFNγ^+^	16.30 ± 5.66	23.73 ± 9.59
	IL-17^+^	0.385 ± 0.262	0.758 ± 0.299

*Distribution of cell populations in peripheral LNs of wt and GHTg mice. Numbers correspond to percentage of positive cells ± SD values (*n* = 3)*.

**Table 4:** Distribution of cell populations in immunized wild-type (wt) and GHTg mice.

	Spleen		Peripheral lymph nodes (LNs)		Mesenteric LNs		Blood	
	wt	GHTg	wt	GHTg	wt	GHTg	wt	GHTg
CD3^+^	33.7 ± 1.2	33.8 ± 4.8	51.7 ± 13.2	43.2 ± 9.7	64.8 ± 3.3	56.1 ± 6.4	28.2 ± 4.2	22.4 ± 2.2
CD4^+^	19.4 ± 2.6	22.5 ± 10.7	27.9 ± 6.5	25.9 ± 5.2	38.2 ± 1.7	36.7 ± 4.1	15.3 ± 2.5	12.7 ± 1.1
CD8^+^	19.0 ± 1.3	17.9 ± 3.4	23.4 ± 6.5	16.4 ± 4.9	28.9 ± 3.1	21.5 ± 4.5	10.5 ± 4.0	8.5 ± 0.9
B220^+^	54.1 ± 5.3	50.8 ± 6.7	**35.4 ± 4.7**	54.0 ± 8.3*	34.8 ± 3.7	41.7 ± 7.0	65.2 ± 3.9	68.8 ± 3.9
NK1^+^	1.9 ± 0.7	1.5 ± 0.5	0.9 ± 0.3	1.0 ± 0.2	0.8 ± 0.1	1.2 ± 0.1	2.5 ± 0.8	2.6 ± 0.9
CD11b^+^	5.3 ± 1.9	6.6 ± 3.7	4.7 ± 1.3	6.5 ± 2.0	7.7 ± 2.3	13.1 ± 2.0	20.5 ± 5.6	24.4 ± 7.2
GR1^+^	5.5 ± 1.5	7.5 ± 3.8	6.6 ± 1.3	8.2 ± 0.9	8.0 ± 1.6	12.4 ± 1.0	15.8 ± 4.0	17.5 ± 5.0
F4/80^+^	4.7 ± 0.8	6.1 ± 2.0	0.9 ± 0.4	1.1 ± 0.8	2.9 ± 1.4	5.4 ± 0.8	6.7 ± 3.0	9.8 ± 5.8

*Distribution of cell populations in blood, spleen, and peripheral LNs of collagen type II-immunized wt and GHTg mice. Numbers correspond to percentage of positive cells ± SD values (*n* = 3). Bold numbers highlight significant differences between GHTg and control mice, as indicated*.

Student’s t-test, *p < 0.05.

**Table 5:** Distribution of CD3^+^/CD4^+^ and CD3^+^/CD8^+^ cell populations in immunized wild-type (wt) and GHTg mice.

		Peripheral lymph nodes (LNs)	
		wt	GHTg
CD4^+^	Foxp3^+^	10.80 ± 1.99	13.55 ± 0.35
	IFNγ^+^	**14.77 ± 3.74**	10.45 ± 2.76*
	IL-17^+^	0.2267 ± 0.0115	0.3850 ± 0.0636
CD8^+^	IFNγ^+^	**19.23 ± 2.003**	13.45 ± 0.2121*
	IL-17^+^	0.3667 ± 0.155	0.28 ± 0.0424

*Distribution of cell populations in peripheral LNs of collagen type II-immunized wt and GHTg mice. Numbers correspond to percentage of positive cells ± SD values (*n* = 3). Bold numbers highlight significant differences between GHTg and control mice, as indicated*.

Student’s t-test, *p < 0.05.

**Figure 4:**
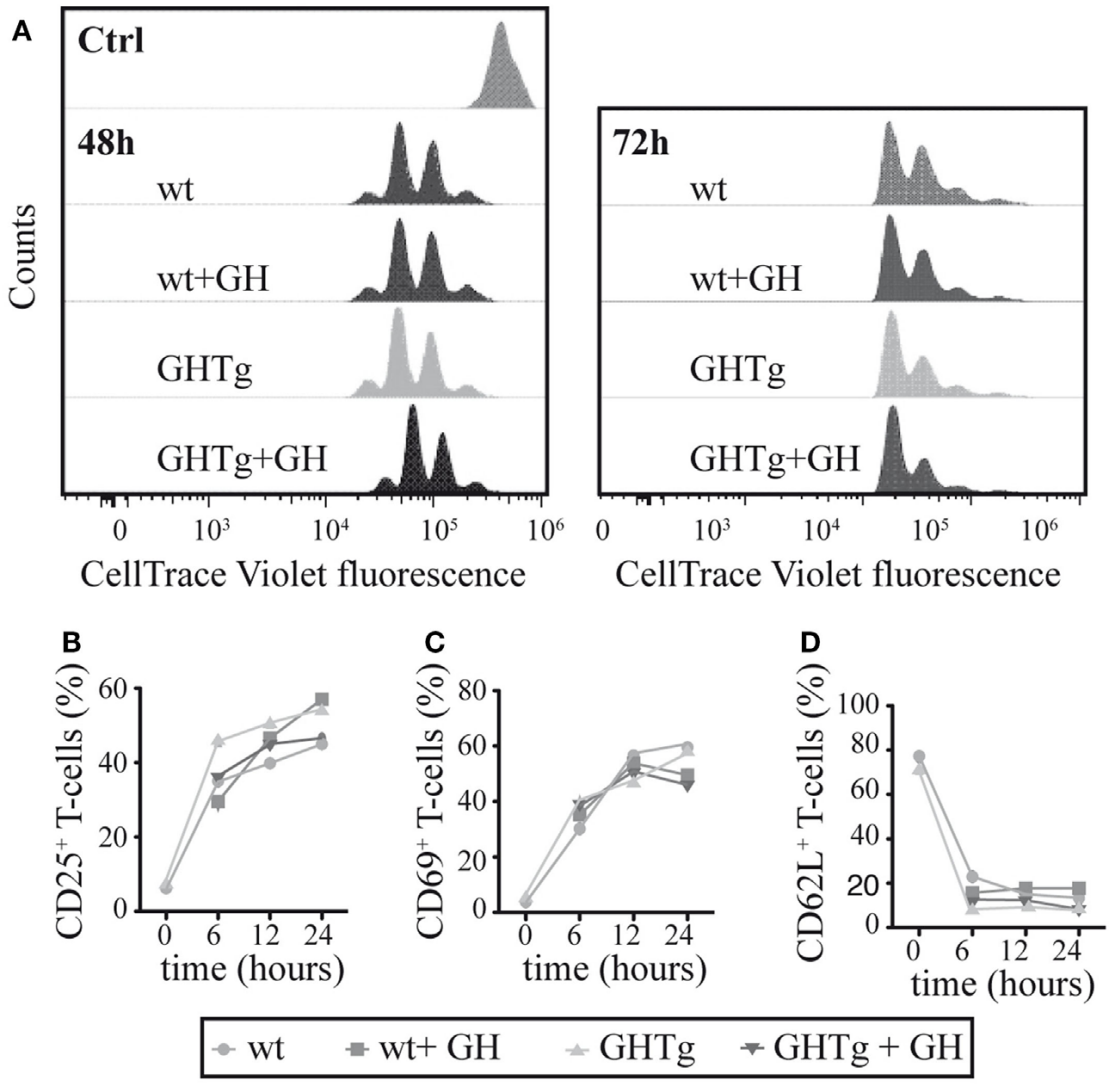
Growth hormone (GH) affects neither bone marrow dendritic cell (BM-DC) antigen presentation nor T cell activation. **(A)** OT-II mouse CD4^+^ splenocytes were stained with CellTrace Violet and co-cultured with bone marrow-derived wild-type (wt) or GHTg dendritic cells (BM-DC), alone or with rhGH. Cells were harvested after 48 or 72 h and analyzed by flow cytometry. A representative experiment is shown of three performed. wt cells at *t* = 0 were used as control. CD4^+^ cells from similar co-cultures were analyzed by flow cytometry at 0, 6, 12, and 24 h for activation markers CD25 **(B)**, CD69 **(C)**, and CD62L **(D)**. One representative experiment is shown of three performed.

To evaluate the GH effect on antigen presentation to T cells, we treated BM-DC derived from GHTg mice or wt controls *in vitro* with LPS and loaded the cells with OVA_323–339_-peptide. Cells were then mixed, alone or with rhGH, with OT-II mouse CD4^+^ cells purified by negative selection and labeled with CellTrace Violet. Flow cytometry detection of cell tracker dilution after 48 and 72 h co-culture showed similar OT-II T cell proliferation in all conditions analyzed (Figure 4A); no differences were observed in CD4^+^ T cell activation markers (CD25, CD69, CD62L) (Figures 4B–D). These results suggest that antigen-presenting cells from GHTg and control littermates have similar antigen-presenting capacity to CD4^+^ T cells and that exogenous rhGH does not affect responder CD4^+^ T cell proliferation.

### GH Regulates the Th17 and Th1 Profiles

**Figure 5:**
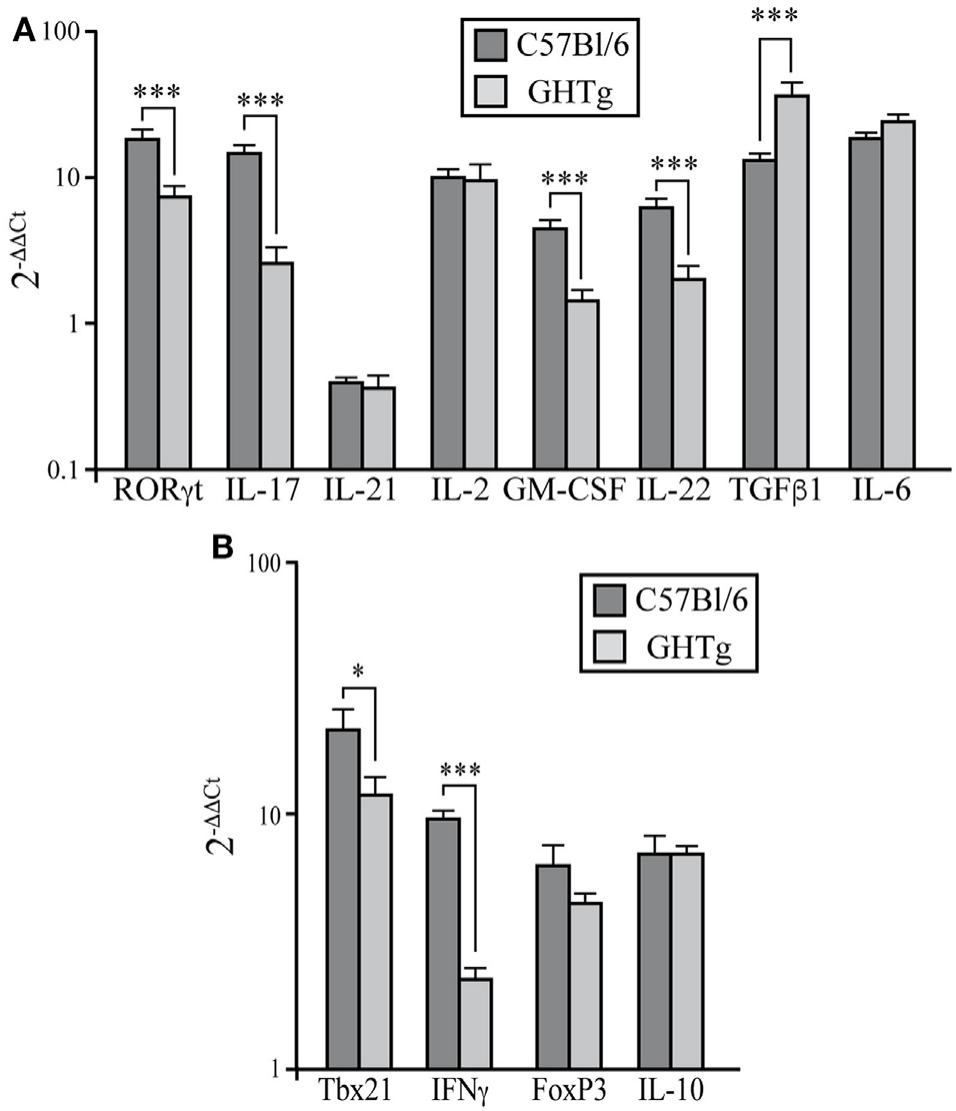
Regulation of pathogenic Th17 profile in collagen-induced arthritis-induced GHTg mice. **(A)** Expression of characteristic Th17 transcription factors and cytokines in peripheral lymph nodes of collagen-immunized wild-type (wt) and GHTg mice. **(B)** Expression of transcription factors and cytokines associated to Th1 and Treg cell responses. Cytokine and chemokine mRNA from arthritic mice was quantified by qRT-PCR. Expression is represented as 2^−ΔΔCt^ by normalization to β-actin and the levels of each cytokine in spleen of wt mice; mean ± SD, Student’s *t*-test (*n* = 12); *p ≤ 0.05; **p ≤ 0.01; ***p ≤ 0.001.

Rheumatoid arthritis has long been considered a Th1-mediated disease, but considerable evidence shows a key role for proinflammatory Th17 cells in inflammation and joint destruction (24, 25). IL-17 levels are high in serum and synovial tissue of RA patients (26). IL-17-deficient allogenic bone marrow transplant in mice prevents CIA and reduces disease severity (27). We thus characterized the Th17 profile in CIA mouse LNs at day 37 post-inoculation. Immunized GHTg mice showed a significant reduction in RORγt, IL-17, GM-CSF, and IL-22 mRNA expression compared to control arthritic littermates, whereas there were no differences in IL-21, IL-6, or IL-2 mRNA levels in peripheral LNs (Figure 5A). These results indicate that GH reduced the pathogenic profile of the Th17 response. In arthritic GHTg vs. wt mice, we also found significantly lower levels of Tbx21 mRNA, the master regulator of the Th1 subset (Figure 5B). These data correlated with a marked reduction in IFNγ mRNA levels in GHTg relative to wt mice. We also found significant higher levels of TGFβ in GHTg mice (Figure 5A), although we did not observed differences in FoxP3 and in IL-10 mRNA levels between the two mouse groups (Figure 5B). We nonetheless observed a tendency to higher FoxP3/RORγT ratio when comparing GHTg (0.59 ± 0.06) with controls (0.32 ± 0.05). These results confirmed the milder disease observed in GHTg mice and support the beneficial effect of GH.

### Therapeutic GH Administration Reduces Disease Severity in CIA Mice

**Figure 6:**
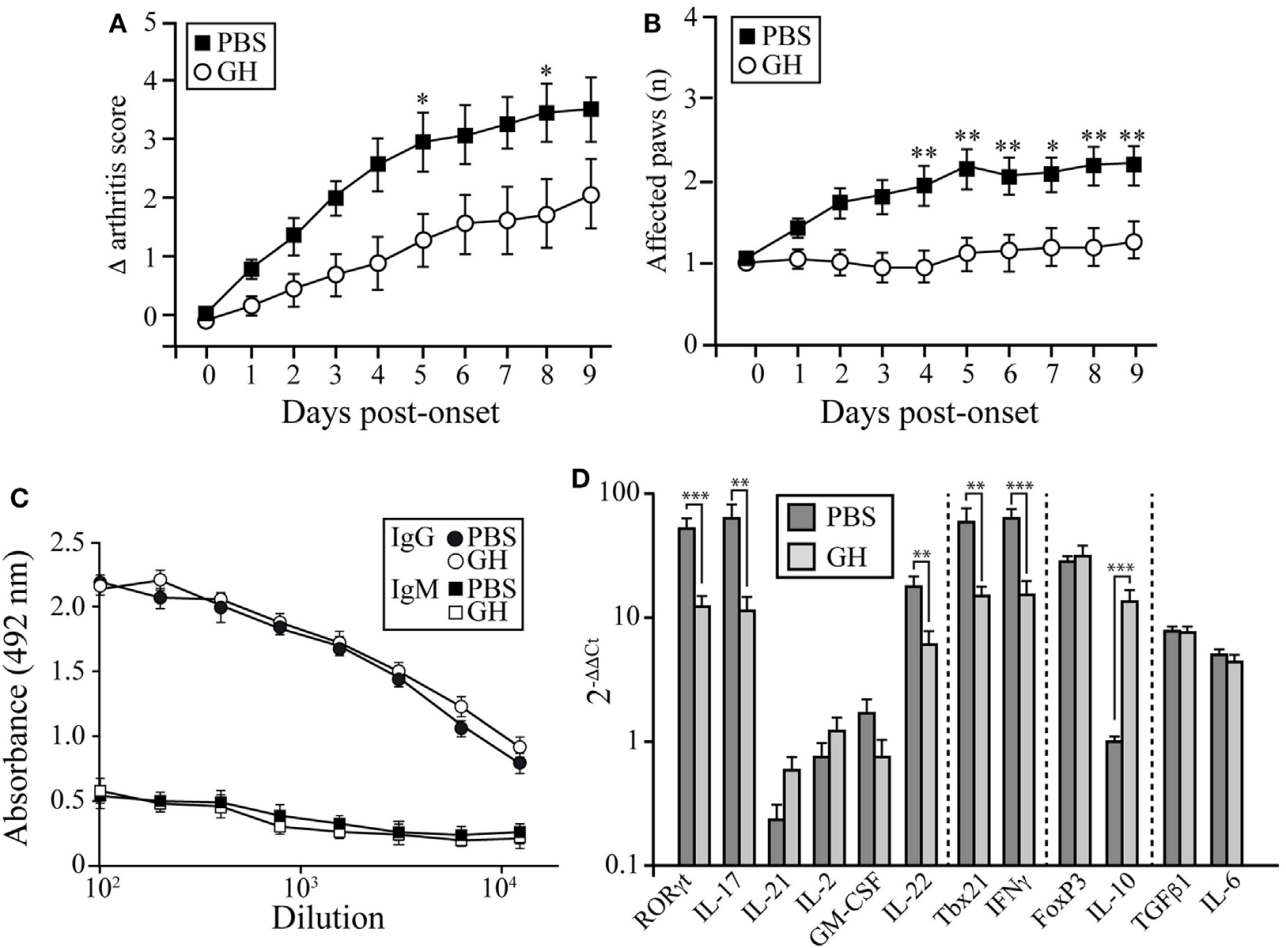
Effect of therapeutic rhGH administration on arthritic mice. Two-month-old DBA/1J mice were immunized with chicken type II collagen. **(A)** On appearance of the first signs of collagen-induced arthritis (score 2 = treatment day 0), affected mice were separated into two groups; one received a daily rhGH injection (2 µg/ml, 200 µl) until day 9, and the other received PBS as control. Clinical scores are expressed as the increase (Δ) from the initial score at *t* = 0 (score 2). Data shown as mean ± SEM. Student’s *t*-test (*n* = 12); *p ≤ 0.05. **(B)** Mean number of affected paws in arthritic mice as in **(A)**. Data shown as mean ± SEM. Student’s *t*-test (*n* = 12); *p ≤ 0.05. **(C)** Total anti-collagen type II IgM and IgG were measured in mouse sera by ELISA at the end of the experiment (day 9 post-treatment) of vehicle- (PBS, black line) or rhGH- (gray line) treated arthritic mice as in **(A)**. The figure shows mean absorbance at 492 nm ± SEM (*n* = 12). **(D)** Expression of characteristic Th17, Th1, and Treg transcription factors and cytokines in peripheral lymph nodes of mice, normalized to β-actin and expressed as 2^−ΔΔCt^. Data shown as mean ± SD. Student’s *t*-test **p ≤ 0.01; ***p ≤ 0.001.

We used the CIA model in DBA/1J mice to assess the potential therapeutic use of GH administration. Arthritis was induced by i.d. injections of collagen type II into 2-month-old mice. On appearance of the first CIA signs (score ~2 = day 0), affected mice were divided into two groups; one received a daily s.c. GH injection until day 10 and the other received PBS as control. CIA progression was delayed in GH-relative to PBS-treated arthritic mice (Figure 6A); this effect was observed as early as 2 days after initiation of treatment and reached a maximum at 6 days. The number of affected paws increased with time in PBS-treated arthritic mice, but GH-treated mice showed no variation (Figure 6B). GH treatment did not alter anti-collagen IgM and IgG antibody levels in arthritic DBA/1J mouse serum (Figure 6C). These data suggest that, in addition to its role in the induction phase of the disease, GH can hinder experimental arthritis progression.

**Figure 7:**
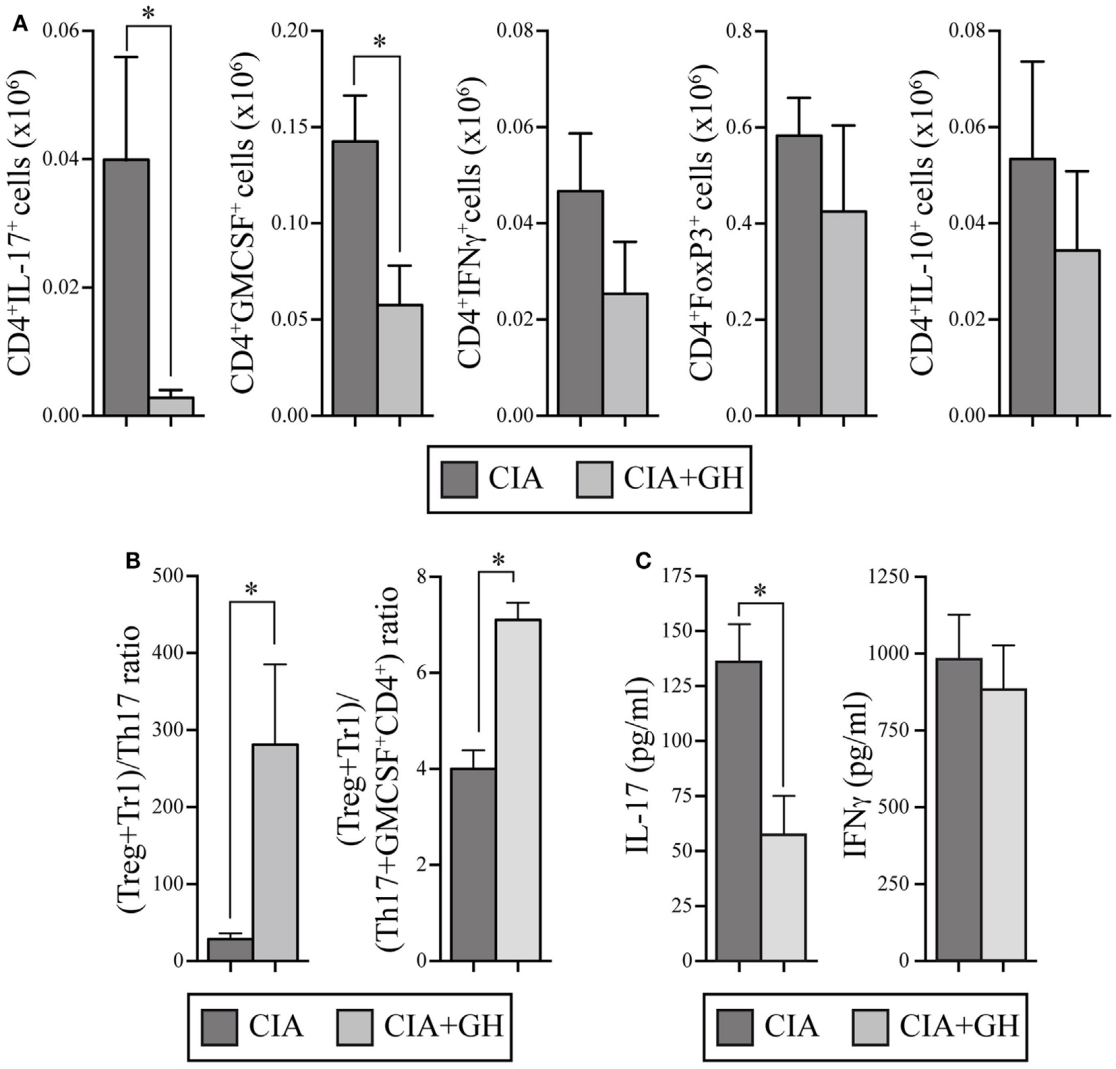
rhGH treatment of arthritic DBA/1J mice modulates the inflammatory cytokine profile in lymph nodes (LNs). **(A)** Quantitation of total number of cytokine-producing CD4^+^ T cells (IL-17, GM-CSF, IFNγ, and IL-10) and regulatory CD4^+^ T cells (FoxP3^+^) from a mixture of popliteal and inguinal LN cells. Data are the mean ± SEM of two different experiments [controls *n* = 10; growth hormone (GH)-treated *n* = 11]. Mann–Whitney *U*-test *p ≤ 0.05. **(B)** Ratios of (Tregs + Tr1)/Th17 cells (left) and of (Tregs + Tr1)/(Th17 + GM-CSF^+^ CD4^+^) cells (right) from popliteal and inguinal LNs of DBA/1J mice as in **(A)**. Mann–Whitney *U*-test *p ≤ 0.05: Tregs (CD4^+^FoxP3^+^ cells); Tr1 (CD4^+^IL-10^+^ cells); Th17 (CD4^+^IL-17^+^ cells). **(C)** Levels of IL-17 and IFNγ in culture supernatants of cells isolated from LNs in **(A)** and activated *in vitro* (48 h) with anti-CD3 mAb. Data shown as mean ± SD. Student’s *t*-test *p ≤ 0.05.

Growth hormone treatment reduced RORγt, IL-17, and IL-22 mRNA expression in LNs at day 10 (Figure 6D). We also detected lower Tbx21 and IFNγ levels in GH- compared to PBS-treated CIA mice, whereas IL-10 levels increased (Figure 6D). When, in the same samples, the expression levels of chemokines were evaluated, we found in GH-treated mice, similar levels of homeostatic chemokines, CCL21 and CXCL12, and a significant reduction of the inflammatory chemokine CCL20 (Figure S1 in Supplementary Material). CCL20 is a key chemokine for Th17 cell movement (28). These data, in line with the results for GHTg arthritic mice, suggested that exogenous rhGH promoted Th17 cell repolarization to a non-pathogenic profile or, alter natively, blocked Th17 polarization to a Th1 phenotype. The analysis of these cells in the LNs of GH-treated CIA mice confirmed these observations. Among CD4^+^ T cells, GH treatment reduced the number of IL-17-expressing cells from 0.040 ± 0.02 × 10^6^ to 0.003 ± 0.01 × 10^6^ cells and of GM-CSF expressing cells from 0.14 ± 0.02 × 10^6^ to 0.06 ± 0.02 × 10^6^ cells in arthritic mice (Figure 7A). In the same experiments, we did not detect significant differences in CD4^+^IFNγ^+^, CD4^+^FoxP3^+^, and CD4^+^IL10^+^ cells (Figure 7A), although a more detailed analysis of the data revealed better regulatory vs. inflammatory balance in GH-treated mice (Figure 7B). In accordance, ELISA data of cytokine production in culture supernatants of anti-CD3 activated cells isolated from these LN showed a GH-mediated significant reduction of IL-17 (56.33 ± 19.5 pg/ml in GH-treated vs. 133.85 ± 17.5 pg/ml in controls) and no significant differences in IFNγ (863.58 ± 157 pg/ml in GH-treated vs. 974.95 ± 107 pg/ml in controls) levels compared to controls (Figure 7C). Together, these data could determine the anti-inflammatory microenvironment triggered by GH, supporting the therapeutic use of GH.

## Discussion

Chronic inflammation underlies many pathologies of clinical importance, including cancer, atherosclerosis, and rheumatic diseases. RA is a systemic autoimmune disease characterized by non-organ-specific autoantibody production and chronic inflammation of synovial tissues, leading to cartilage and bone destruction (29). Current therapies, such as cytokine antagonists, have shown great promise in treating patients (30), although they target the inflammation phase and do not address the initiation or resolution phases, that is, those situations in which the balance between effector and regulatory responses are disrupted (31).

There is evidence that the immune system is greatly influenced by other physiological systems such as the endocrine and neuroendocrine systems (32). GH is produced not only by the pituitary gland but also by lymphoid organs such as the thymus, the spleen, and immune cells in the periphery (33), and the GH receptor is expressed on most leukocyte subpopulations (34). Here, we used GHTg mice, in which bGH expression is ubiquitous and sustained, to analyze GH effects on induction of experimental arthritis. In a CIA model, we showed that GHTg mice were partially protected compared to wt arthritic littermates. GHTg mice showed delayed disease onset and reduced severity. Pannus, cartilage damage, and bone resorption were detected only in wt CIA mice, whereas we found no sign of joint degradation in GHTg mice at day 30 post-inoculation. It is well known that GH regulates both bone growth and remodeling, although it is unclear whether they are effects mediated directly or through IGF-I signaling (35). GH modulates both osteoblasts and osteoclasts differentiation and activity (36–38). Although we have not analyzed the activity of osteoblasts in these mice, we did not detect osteoclasts in the joint of collagen-immunized GHTg mice, suggesting that additional causes might also contribute to the resistance to arthritis development in these mice. A previous report described a spontaneous autoimmune-like disorder accompanied by alterations in joints of 6-month-old GHTg mice (39). The phenotype in the joints of these mice includes disorganization of zonal structure, profound alterations in chondrocyte growth/differentiation processes and cartilage damage, and it is complemented by increased levels of auto-anti-DNA antibodies compared to control littermates. The authors, however, rarely found inflammatory infiltrates in synovial tissues indicating that the symptoms detected are more related to osteoarthritis related to bone overgrowth rather than to RA. We did not observe visceromegaly or changes in weight or in length of DBA/1J mice treated with exogenous rhGH, but as it is known that GHTg mice develop giantism (18, 40), we cannot discard potential side-effects in treatments longer than those used in this study.

In accordance with previous observations (13, 15), we detected no major differences in any of the circulating immune cell populations analyzed in basal conditions, although we observed a reduced percentage of CD3^+^IFNγ^+^ cells in immunized GHTg mice and a marked increase in the percentage of B220^+^ cells in LNs of GHTg mice that was maintained after collagen immunization. We nonetheless noticed a marked reduction in anti-chicken collagen II antibodies in the sera of immunized GHTg mice, although no intrinsic defects were seen in *in vitro* B cell activation by either anti-IgM antibodies or LPS. We also ruled out defects in the ability of GHTg mouse BM-DC to present antigen and activate T cells *in vitro*, although the percentage of activated circulating CD4^+^ cells in GHTg mice was lower than in wt arthritic mice.

Autoreactive effector CD4^+^ T cells, specifically the IFNγ-producing Th17/Th1 and non-classical Th1 cells, are associated with RA pathogenesis (41). We detected lower IFNγ mRNA levels, reduced CD3^+^IFNγ^+^ cells and a significant reduction in Tbx21 expression in LNs from GHTg with respect to wt arthritic mice, which is indicative of the protective role of GH in CIA. In these mice, RORγt, IL-17, GM-CSF, and IL-22 mRNA expression was also significantly reduced, indicating a reduction in the percentage of Th17 cells. A similar expression pattern of cytokines was found in GH-treated arthritic DBA/1J mice. Altogether, these results suggest that GH maintains a non-pathogenic profile of Th17 cells. Th17 cell frequency in peripheral blood correlated directly with disease activity. In RA patients, IL-17 levels are similarly increased in synovial fluid (42) and contribute to sustain RA chronicity (24). There is evidence that Th17 cells are a heterogeneous population composed of several pro- and anti-inflammatory subsets (43). We also observed increased TGFβ1 and no significant variations in IL-6 mRNA levels. TGFβ has been associated with the maintenance of peripheral tolerance by inhibiting proliferation and differentiation of autoreactive T cells (44) and promoting survival of Treg cells (45). Although we found no marked differences in the absolute FoxP3 mRNA levels between GHTg and control mice, immunized GHTg mice had a higher FoxP3/RORγT ratio, concurring to previous results obtained in a model of diabetes type I in NOD-GHTg mice (15). We also found higher ratio of Treg/Th1 cells after GH treatment of arthritic DBA/1J mice compared to controls. IL-6 blockade corrects the imbalance between Th17 and Treg cells in patients with RA (46). In the absence of IL-6, TGFβ do not generate Th17 cells (47–49). Our results thus suggest that GH also favors the immunoregulatory responses.

Development of therapeutic strategies that promote regulatory mechanisms could allow the establishment of robust, sustained resolution of autoimmunity. Moreover, the use of mediators endogenously expressed might reduce the associated side-effects of these treatments. Here, we demonstrate the beneficial effect of GH administration at the first signs of arthritis in collagen-immunized mice, as well as a clear delay in arthritis progression in GHTg compared to control littermates. Several rheumatic diseases are characterized by abnormalities in the GH/IGF-1 paracrine axis (50), and although in adults the relationship between serum GH levels and disease progression is debated (51), juvenile chronic arthritis patients have reduced GH levels (52). In the arthritic DBA/1J model, GH treatment did not affect circulating anti-collagen antibody levels, which suggests that in this model, GH modulates the progression and resolution phases of CIA. The mechanism also involved a GH effect on the immune system, as shown by a significant reduction in IFNγ, IL-17, and IL-22 mRNA levels in LNs of GH-treated CIA mice, with a concomitant increase of IL-10. In contrast of these results, IL-10 expression was not elevated in GHTg mice. Although more experiments are needed to clarify this discrepancy, differences in circulating levels of GH between GHTg and GH-treated DBA/1J mice might cause this difference. Whereas GHTg mice have sustained GH levels in sera (~5 µg/ml), wt mice have a pulsatile and circadian expression of GH that in our DBA/1J model is modified by the exogenous administration of the hormone (2 µg/ml). Acute responses of GH must thus differ in both mouse models as well as how mice are adapted to this circumstance.

Our flow cytometry data on peripheral LNs cells from arthritic DBA/1J mice confirmed the effect of GH treatment on the reduction number of Th17 and CD4^+^GM-CSF^+^ T cells. These data correlated with the significant reduction of CCL20 levels found in LNs of mice treated with the hormone. CCL20 binds to CCR6, an important chemokine receptor for Th17 cells movement (28). CCR6 expression by Th17 cells allows their migration across the endothelial barrier in several autoimmune diseases such as multiple sclerosis (53) or RA (54). The analysis, however, did not show modifications in the number of CD4^+^Foxp3^+^ and CD4^+^IL-10^+^ T cells, discarding the participation of these cells in the increase of IL-10 levels found in GH-treated mice. Mesenchymal stem cells (MSCs) are capable of suppressing the immune response by inhibiting the maturation of dendritic cells and suppressing the function of T lymphocytes, B lymphocytes, and natural killer cells in autoimmune and inflammatory diseases (55). In *in vitro* experiments, MSCs prevent the differentiation of naïve CD4^+^ T cells into Th17 cells and inhibited the production of IL-17, IL-22, IFN-γ, and TNF-α by fully differentiated Th17 cells, inducing the expression of Foxp3 and IL-10 production (56). We observed higher ratio of (Treg + Tr1)/Th17 and of (Treg + Tr1)/(Th17 + GM-CSF^+^CD4^+^) cells in GH-treated arthritic mice compared to controls. Taking into account that MSC express a functional GH receptor (57), we can hypothesize that via mobilization from their reservoirs and/or by affecting their tropism, GH treatment might facilitate the presence of MSC in the LNs, thus favoring IL-10-mediated regulatory responses. By increasing in the LNs the levels of Tregs cells, including CD4^+^IL10^+^ Tr1 cells, and triggering Th17 cell plasticity toward an IL10-driven anti-inflammatory response, adipose-derived MSCs ameliorated established CIA (58). The combination between MSC and IL-10-producing Tr1 cells prevents the development of destructive arthritis (59). Although we did not observe significant differences in the percentage of F4/80^+^IL-10^+^ cells between GH-treated DBA/1J and control mice, MSCs can also modulate macrophage differentiation toward an anti-inflammatory phenotype (60, 61). MSC also induce regulatory DCs in the LNs (62). This subpopulation of DCs are characterized by the secretion of high levels of IL-10 which is essential for the regulation of the immune responses (63). To clarify all these hypotheses, new experiments are actually in progress.

The reduction of Tbx21 levels detected in GH-treated mice also suggest that exogenous GH repolarizes Th17 cells to a less aggressive phenotype and/or blocks the acquisition of a pathogenic profile of this Th17 cells. GH triggers JAK2/STAT5 phosphorylation (64) and STAT5 controls not only Th2 but also Th1 and Th17 differentiation; IL-2 regulates Th1 and Th17 differentiation, which induces STAT5-dependent IL-12Rβ2 and Tbx21 in Th1 cells and IL-17 expression by Th17 cells (65). We can thus hypothesize that GH treatment regulates the expression of genes involved in T cell polarization by altering STAT5 phosphorylation directly or indirectly *via* SOCS expression regulation.

Our results confirm the importance of GH in regulating immune system plasticity, and thus its value as a regulatory mechanism that limits the effector response and maintains immune system homeostasis. Recombinant human GH improves survival and protects against acute lung injury in murine *Staphylococcus aureus* sepsis (66) and protects from acute pancreatitis (67). GH decreases gut inflammation and improves or maintains gut barrier function, which ultimately inhibits development of inflammatory bowel disease (14, 68); co-administration of epidermal growth factor and GH-releasing peptide-6, a GH secretagogue, improves clinical recovery in experimental autoimmune encephalitis (69). GH is thus a candidate for potential treatment of inflammatory disorders and therapy for arthritis and other autoimmune diseases.

## Ethics Statement

Mice were handled according to national and European Union guidelines, and experiments were approved by the Comité Ético de Experimentación Animal, Centro Nacional de Biotecnología and the Regional Government (PROEX 250-16).

## Author Contributions

RV, GC, YJ, ML-S, EG-C, JR-F, JL, and PL performed the experiments and analyzed the data. GC, CM-A, MG, and RPG designed experiments and interpreted the data. RV, GC, YJ, JLP, CM-A, MG, and RPG revised critically the manuscript. RV and MM conceived the idea designed the experiments, interpreted data, and wrote the manuscript. All the authors have revised and approved the final version of the manuscript.

## Conflict of Interest Statement

The authors declare that the research was conducted in the absence of any commercial or financial relationships that could be construed as a potential conflict of interest.
