# Supplementary material for: Inhibitory Role of Growth Hormone in the Induction and Progression Phases of Collagen-Induced Arthritis
Source: Front Immunol. 2018 May 25;9:1165. doi: 10.3389/fimmu.2018.01165 (PMC5980961; doi:10.3389/fimmu.2018.01165)
Supplement: Supplementary file 1 [file Image_1.pdf]

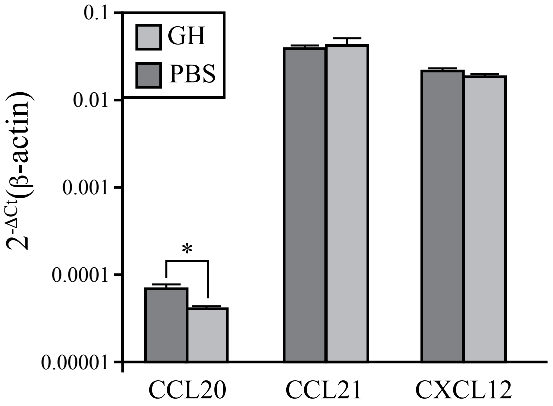

Figure S1 . Effects of rhGH administration the Expression of chemokines in peripheral LNs of arthritic mice. Chemokine mRNA from GH-treated arthritic DBA/1J and control mice was quantified by qRT-PCR. Expression is normalized to b-actin and expressed as $2^{-\Delta Ct}$. Data shown as mean ± SD (*n* = 12). Student's t-test *p ≤0.05.
